# Did universal masking during the COVID-19 pandemic reduce MRSA and MSSA acquisition in the NICU?

**DOI:** 10.1017/ice.2025.10209

**Published:** 2025-08

**Authors:** Meaghan Neary, Kathleen Quan, Thomas Tjoa, Cassiana E. Bittencourt, Susan S. Huang, Cherry Uy

**Affiliations:** 1 Division of Neonatal Medicine, Department of Pediatrics, University of California Irvine Health, Orange, CA, USA; 2 Department of Epidemiology & Infection Prevention, University of California Irvine Health, Orange, CA, USA; 3 Division of Infectious Diseases, University of California Irvine School of Medicine, Irvine CA, USA; 4 Department of Pathology and Laboratory Medicine, University of California Irvine School of Medicine, Irvine, CA, USA

## Abstract

**Objectives:**

To assess whether universal masking during the COVID-19 pandemic reduced neonatal acquisition of *S. aureus*.

**Study design:**

We performed a retrospective cohort study of neonates admitted to a level three regional NICU for three years before and after implementation of universal masking for the COVID-19 pandemic. Multivariable proportional hazards regression models evaluated the effect of masking on time-to-acquisition of methicillin-resistant and methicillin-sensitive *S. aureus* (MRSA and MSSA) while adjusting for fixed and time-varying neonatal characteristics.

**Results:**

We analyzed 2,728 neonates, 1,446 pre-pandemic and 1,282 post-pandemic; 84.9% were inborn, with mean gestational age of 34 weeks and 6 days (SD = 4.2) and mean birthweight of 2,500 grams (SD = 975). The mean number of screening cultures per neonate was 3.07 (SD = 3.31). When adjusting for covariates, universal masking was associated with decreased acquisition of MRSA (hazard ratio =0.43 (95% CI: 0.19–0.99), *p* = 0.04) but not MSSA (HR = 1.27 (95% CI: 00.87–1.85), *p* = 0.21). Among covariates, airway devices and maternal *S. aureus* status were associated with *S. aureus* acquisition.

**Conclusions:**

Universal masking decreased the rate of NICU MRSA acquisition by 60% while MSSA acquisition was unchanged. Masking may reduce MRSA spread via colonized healthcare personnel while MSSA may be more likely to be acquired from parental skin-to-skin contact and was thus unaffected by masking.

## Introduction

*Staphylococcus aureus* is a major source of outbreaks and invasive disease in the neonatal intensive care unit (NICU) where it causes bacteremia, skin and soft tissue infections, omphalitis, osteomyelitis, sepsis, toxic shock syndrome, and necrotizing pneumonia.^1,2^ Invasive *S. aureus* infection in neonates has a reported mortality of 10–25%.^3,4^ One of the most significant risk factors for invasive *S. aureus* disease is prior colonization with up to a 24-fold relative risk of infection in neonates colonized with MRSA.^2^

*S. aureus* colonization is typically concentrated in the nares, as well as skin, axilla, groin, and rectum.^5^ *S. aureus* is a human commensal organism, with nasal colonization in healthy individuals estimated between 10–50%.^2,6^ Transmission of *S. aureus* colonization from parents to neonate is expected within the first several months as the microbiome is established, however there are additional factors within the NICU to consider including transient colonization of healthcare workers, risk of outbreaks, and environmental reservoirs. Extreme prematurity, low birth weight, broken skin barriers and indwelling devices are all risk factors for infection with *S. aureus*, especially in a colonized individual.^7^

Implementing targeted preventative measures, including contact precautions (isolating patients and wearing gown and gloves during patient contact) and strict compliance with hand hygiene and environmental decontamination can decrease transmission of *S. aureus* and reduce risk of infection.^7,8^ In recent years, there has also been a focus on targeted decolonization of patients^9^, parents^10^ and healthcare personnel.^11^ However, despite these measures, contemporary outbreaks of MRSA within the NICU setting continue to be described.^11–13^

Lacey et al.^14^ demonstrated a significant reduction in transient colonization with MRSA in healthcare workers wearing masks while caring for patients colonized and infected with MRSA, however this is not a common practice. Masking is frequently employed to minimize risk of transmission of a variety of other infectious organisms, but it is not commonly employed in management of MRSA within the hospital setting. The predilection for *S. aureus* to reside in the anterior nares raises the question of whether masking may further minimize the spread in high-risk settings.

In March 2020, due to the COVID-19 pandemic, our institution implemented universal masking for all healthcare personnel and visitors, including parents of NICU patients. This change in policy allowed the opportunity to analyze the effect of masking on the acquisition of *S. aureus* within our NICU population.

## Methods

This retrospective cohort study was conducted at a level III regional NICU with 50% single bed rooms at the University of California, Irvine (UCI) Medical Center to assess the impact of universal masking on neonatal acquisition of MRSA and MSSA. Data were collected for the 39-month period before (January 2017–March 2020) and the 36-month period after (April 2020–March 2023) universal masking was instituted due to the COVID-19 pandemic. This study was conducted as a quality improvement project. As an operational project, human subjects approval was not required but was obtained from the UCI Institutional Review Board.

Beginning in April 2020, all medical center staff and visitors were required to wear masks in both patient care and non-patient care areas of the hospital, and adherence to the mask policy was extremely high. A hospital-wide symptom screening process was implemented to minimize risk of COVID-19-positive individuals entering the hospital as visitors or employees. During this time, only two designated visitors (usually parents) were allowed per neonate for the duration of their NICU stay. This was a change in policy from the pre-pandemic period, when each infant could have an additional four designated visitors. Routine precautions in the NICU remained the same before and during the pandemic masking period, including (1) requiring all staff and visitors to perform hand and forearm disinfection and ensuring arms were bare below the elbow prior to NICU entry, (2) screening all patients on admission for MRSA (using a process that also identified and reported MSSA carriage), (3) isolating neonates positive for MRSA or other multi-drug resistant organisms, and (4) decolonizing infants identified to be MRSA carriers as well as those positive for MSSA with a nasal device in place (continuous positive airway pressure (bCPAP), or nasal cannula). Decolonization was performed with intranasal mupirocin for all affected neonates and chlorhexidine baths in qualifying older infants in both the pre-pandemic period and pandemic period.

California state mandates that hospital admissions meeting certain criteria (surgical, dialysis, ICU, nursing home resident, and recent admission in the past 30 days) be screened for MRSA using bilateral nasal swabs. Instead, UCI screens all admissions for both MRSA and MSSA. In addition, our NICU performed additional screening of infants, initially monthly, and then transitioning to weekly by the latter half of 2019.

Screening swabs of nares and skin (axilla and groin) from babies were cultured for MSSA using sheep blood agar and for MRSA using chromogenic agar (Spectra MRSA, ThermoFisher Scientific, Waltham, MA). The *S. aureus* identification was confirmed using matrix-assisted laser desorption ionization-time of flight mass spectrometry (MALDI-ToF-MS). Standardized susceptibility was performed using the VITAK-2 GP-67 card (bioMérieux, Marcy-l’Etoile, France). MRSA and MSSA results from admission screening and clinical tests throughout hospitalization were obtained from the electronic health record for all inborn and outborn neonates, as well as inpatient mothers. Inborn infants refers to infants who were born at UCI Medical Center, outborn infants refers to those who were born at other hospitals and transferred to the NICU at UCI. A positive culture after hospital day 3 was deemed to be a neonatal acquisition event for *S. aureus.* Neonatal characteristics were obtained from a departmental neonatal database with chart review performed for missing data. Collected variables included dates of admission and discharge, birthweight, gestational age, mode of delivery, inborn or outborn status, presence of a central line (umbilical catheters, peripherally inserted central catheters, or surgically placed central venous catheter), presence of an airway device (bCPAP, endotracheal tube, or tracheostomy), and whether or not the neonate underwent surgery during the hospitalization.

The primary outcomes were incident MRSA and MSSA cultures, analyzed separately. Bivariate analyses were performed using chi-square tests for categorical variables and t-tests for continuous variables. Multivariable proportional hazards regression models were performed to evaluate the effect of period (masking versus not masking) on time to acquisition of MRSA or MSSA while adjusting for fixed covariates (gestational age, birthweight, sex, maternal MRSA and MSSA carriage in the year prior to delivery) and time-varying covariates (airway devices and number of screening cultures by day of hospitalization). Since neonates experienced differing amounts of weekly MRSA surveillance tests due to variable lengths-of-stay and different screening policies across the study, accounting for the hospital day and number of screening samples as time-varying covariate was important. Maternal MRSA and MSSA carriage were input as categorical variables (positive, negative, missing). If variables were found to be collinear, one variable was retained based upon clinical meaningfulness. Significance was determined at *α* = 0.05. All analyses were performed using SAS 9.4 (Cary, NC).

## Results

A total of 2,728 neonates were included during the study period, 1,446 neonates from pre-pandemic and 1,282 during the pandemic. Among all neonates, 84.9% (2,316) were inborn, with a mean gestational age of 34 weeks 6 days (SD = 4.2) and birthweight of 2,500 grams (SD = 975). Neonatal characteristics were similar between the two periods (Table 1), although neonates were slightly younger and had more central lines pre-pandemic.

**Table 1.** Neonatal characteristics, before and after initiation of universal masking due to the COVID-19 pandemic

	All neonates *n* (%)	Pre-Pandemic January 2017–March 2020 *n* (%)	Pandemic masking period April 2020–March 2023 *n* (%)	*P*-value^1^
Number of neonates	2728	1,446	1,282	
Gestational age				
≥ 37 weeks	1,183 (43.4%)	621 (43.0%)	562 (43.8%)	0.01
32 to < 37 weeks	996 (36.5%)	504 (34.9%)	492 (38.4%)	
28 to < 32 weeks	350 (12.8%)	197 (13.6%)	153 (11.9 %)	
Less than 28 weeks	199 (7.3%)	124 (8.6%)	75 (5.8%)	
Birth weight				
Greater than 2500 grams	1,415 (51.9%)	724 (50.1%)	691 (53.9%)	0.004
1500 to < 2500 grams	833 (30.5%)	436 (30.2%)	397 (31.0%)	
1000 to < 1500 grams	266 (9.8%)	149 (10.3%)	117 (9.1%)	
Less than 1000 grams	214 (7.8%)	137 (9.5%)	77 (6.0%)	
Male	1,478 (54.2%)	764 (52.8%)	714 (55.7%)	0.13
Inborn	2,316 (84.9%)	1,221(84.4%)	1,095 (85.4%)	0.48
Born via cesarean section	1,575 (57.7%)	850(58.8%)	725 (56.6%)	0.24
Expired	62 (2.3%)	32 (2.2%)	30 (2.3%)	0.82
Length-of-stay in days, mean (SD)	21.7 (28.0)	22.6 (29.3)	20.7 (26.5)	0.07
Surgery	148 (5.4%)	78 (5.4%)	70 (5.5%)	0.94
Central line	1,024 (37.5%)	577 (39.9%)	447 (34.9%)	0.007
Duration of central line in days, mean (SD)	13.1 (20.5)	12.9 (18.9)	13.3 (22.3)	0.73
Airway devices				
bCPAP^2^	963 (35.3%)	518 (35.8%)	445 (34.7%)	0.54
ETT^3^	643 (23.6%)	359 (24.8%)	284 (22.2%)	0.10
Tracheostomy	15 (0.55%)	5 (0.35%)	10 (0.78%)	0.13
Neonates using airway device^4^	1,197 (43.9%)	642 (44.4%)	555 (43.3%)	0.56
Duration of airway device in days, mean (SD)	16.1 (25.3)	16.8 (25)	15.3 (25.5)	0.32
Mean screening tests per patient, M (SD)	3.07 (3.31)	2.41 (2.52)	3.86 (3.91)	< 0.001
Neonates with MSSA found in first 3 days of hospital stay	39 (1.4%)	18 (1.2%)	21 (1.6%)	0.42
Total neonates acquiring MSSA after hospital day 3^5^	152 (5.6%)	61 (4.2%)	91 (7.1%)	0.001
Neonates with MRSA found in first 3 days of hospital stay	4 (0.2%)	2 (0.1%)	2 (0.2%)	0.99
Total neonates acquiring MRSA after hospital day 3^6^	32 (1.2%)	21 (1.5%)	11 (0.9%)	0.15
Number of mothers with MSSA in 1 year prior to birth^7^	347(15.0%)	157(12.8%)	190 (17.3%)	0.002
Number of mothers with MRSA in 1 year prior to birth^7^	31 (1.3%)	15 (1.2%)	16 (1.5%)	0.62

1 Reflects chi-square for categorical variables and t-test for continuous variables comparing pre-pandemic to post pandemic periods.

2 Bubble continuous positive airway pressure device.

3 Endotracheal tube.

4 Any airway device refers to the patients who ever had bCPAP, ETT, or tracheostomy in place during hospitalization, some infants had multiple airway devices during their NICU stay and were only counted once in regards to this total.

5 Denominator reflects 2,689 infants who did not have MSSA in first 3 days of hospital stay.

6 Denominator reflects 2,724 infants who did not have MRSA in first 3 days of hospital stay.

7 Data available for 2,319 mothers, 1,223 pre-pandemic and 1,096 during the pandemic masking period.

A total of 184 neonates acquired *S. aureus* during the study period. For MRSA there were 32 acquisition events, 21 pre-pandemic and 11 during the universal masking pandemic period. The rate of MRSA acquisition per 1000 patient days decreased from 0.64 pre-pandemic to 0.37 during universal masking.

For MSSA there were 152 acquisition events, 61 in the pre-pandemic period and 91 in the universal masking pandemic period. In contrast to MRSA, the rate of MSSA per 1,000 patient days increased from 1.87 prior to pandemic masking period to 3.43 during universal masking, although the number of screening cultures per neonate was significantly greater during the pandemic (3.86 (SD = 3.91) compared to pre-pandemic (2.41 (SD = 2.52), *p*-value <0.0001) due to a change in screening policy. This change in screening frequency was accounted for as a time-varying covariate in multivariable models below.

Bivariate analyses (Table 2) showed that maternal MSSA carriage was significantly associated with *S. aureus* acquisition for MSSA. The use of certain airway devices was also associated with *S. aureus* acquisition for both MRSA and MSSA. Maternal MRSA positivity was associated with a hazard ratio of 5.00 (95% confidence interval 0.66–37.74, *p* = 0.12) for neonatal MRSA acquisition, and maternal MSSA positivity was associated with a hazard ratio of 2.55 (95% confidence interval 1.72–3.77, *p* < 0.001) for neonatal MSSA acquisition. Unadjusted results showed that the pandemic period was associated with higher MSSA acquisition prior to accounting for the greater frequency of testing during this period using multivariable modeling.

**Table 2.** Bivariate (unadjusted) associations between universal masking, neonatal characteristics and *S. aureus* acquisition

	Methicillin-resistant *Staphylococcus aureus (*MRSA)		Methicillin-susceptible *Staphylococcus aureus (*MSSA)	
	Hazard ratio (95% Confidence Interval)	P-Value	Hazard Ratio (95% Confidence Interval)	P-Value
Pandemic masking period^1^	0.62 (0.29–1.32)	0.21	1.96 (1.42–2.71)	< 0.001
Number of screening tests sent	1.13 (0.92–1.38)	0.25	1.37 (1.21–1.57)	< 0.001
Gestational age (referent ≥37 weeks)				
32 to < 37 weeks	1.20 (0.33–4.38)	0.78	0.97 (0.56–1.67)	0.91
28 to < 32 weeks	0.99 (0.27–3.69)	0.99	1.13 (0.65–1.97)	0.67
< 28 weeks	1.22 (0.31–4.76)	0.77	1.13 (0.62–2.06)	0.68
Birthweight (referent ≥ 2500 grams)				
1500 to < 2500 g	2.19 (0.62–7.69)	0.22	1.00 (0.62–1.62)	0.99
1000 to < 1500 g	1.00 (0.23–4.26)	0.99	1.39 (0.84–2.28)	0.20
< 1000 g	2.09 (0.56–7.88)	0.27	0.87 (0.50–1.50)	0.61
Male sex	0.95 (0.47–1.91)	0.89	0.94 (0.68–1.30)	0.71
Inborn	1.57 (0.64–3.86)	0.32	1.24 (0.81–1.91)	0.33
Born via Cesarean section	1.95 (0.75–5.08)	0.17	1.00 (0.70–1.43)	0.98
Length of stay	1.01 (0.99–1.02)	0.16	1.00 (0.99–1.01)	0.89
Surgery	0.83 (0.28–2.45)	0.74	1.32 (0.87–2.02)	0.19
Central line	1.28 (0.50–3.31)	0.61	1.16 (0.78–1.72))	0.47
bCPAP^2^	2.61 (0.89–7.64)	0.08	1.27 (0.87–1.86)	0.22
ETT^3^	1.08 (0.52–2.24)	0.84	1.56 (1.11–2.18)	0.01
Tracheostomy	1.88 (0.25–13.95)	0.54	1.16 (0.37–3.66)	0.80
Any respiratory device	3.68 (0.86–15.87)	0.08	1.53 (0.98–2.40)	0.06
Maternal *S. aureus*^4^	5.00 (0.66–37.74)	0.12	2.55 (1.72–3.77)	< 0.001

1 Pandemic masking period (April 2020–March 2023) compared to pre-pandemic period (January 2017–March 2020) (referent).

2 bCPAP refers to bubble continuous positive airway pressure device in place.

3 Endotracheal tube.

4 Maternal MRSA or MSSA status in the one year prior to delivery.

In multivariable models (Table 3), the universal masking pandemic period was significantly associated with decreased neonatal acquisition of MRSA (HR = 0.43 (95% confidence interval 0.19–0.99), *p* = 0.04), but not acquisition of MSSA (HR = 1.27 (95% confidence interval0.87–1.85), *p* = 0.21).The presence of an airway device was significantly associated with neonatal MRSA acquisition (HR = 4.63 (95% confidence interval 1.01–21.09), *p* = 0.05). Maternal MSSA status also remained significantly associated with neonatal MSSA acquisition (HR = 2.32, (1.60–3.37), *p* =< 0.001). Birthweight and gestational age were found to be collinear (Pearson Rho = 0.86, *p* < 0.001). Gestational age, as a categorical variable, was retained in the final model.

**Table 3.** Multivariable associations between universal masking, neonatal characteristics, and *S. aureus* acquisition

	MRSA		MSSA	
	Hazard ratio (95% confidence interval)	*P*-value	Hazard ratio (95% confidence interval)	*P*-value
Pandemic masking period^1^	0.43 (0.19–0.99)	0.04	1.27 (0.87–1.85)	0.21
Number of screening tests sent	1.24 (0.97–1.57)	0.08	1.31 (1.14–1.51)	< 0.001
Gestational age (referent ≥ 37 weeks)		0.65		0.97
32 to < 37 weeks	0.89 (0.23–3.49)		1.08 (0.59–1.99)	
28 to < 32 weeks	0.81 (0.21–3.08)		0.99 (0.56–1.76)	
< 28 weeks	0.89 (0.23–3.49)		1.08 (0.59–1.99)	
Male sex	1.07 (0.52–2.18)	0.86	1.02 (0.74–1.42)	0.89
Maternal MSSA/MRSA status^2^	4.42 (0.56–34.75)	0.16	2.32 (1.6–3.37)	< 0.001
Presence of airway devices^3^	4.63 (1.01–21.09)	0.05	1.43 (0.88–2.33)	0.15

1 Pandemic masking period (April 2020–March 2023) compared to pre-pandemic period (January 2017–March 2020) (referent).

2 Maternal MRSA or MSSA status in the one year prior to delivery.

3 Includes presence of CPAP (continuous positive airway pressure device), endotracheal tube, or tracheostomy.

## Discussion

In a Level III NICU, universal masking of staff and parents during the pandemic masking period significantly reduced neonatal MRSA acquisition, but not MSSA. These results are similar to those published by McNeil et al.^16^ who found that the colonization of healthy children with MRSA decreased significantly during the pandemic, while MSSA colonization increased. Neonates are rarely colonized by *S. aureus* at birth, and exposure to care providers (both parents and healthcare workers) serves as the main source of acquisition of this human commensal, along with the lesser route of environmental fomites. This selective protective effect of masking on MRSA, but not MSSA acquisition, may be due to differential pathways for neonatal acquisition. As a healthcare-associated pathogen, MRSA was more likely to be acquired from colonized healthcare personnel who may have acquired it from colonized patients.^11,15^ Our center has previously published our own rate of healthcare staff colonization as 3.4%.^11^ Other studies have demonstrated similar numbers, with the proportion of healthcare workers colonized by MRSA often found to be over 4%.^17,18^ The maternal colonization rate in our population was 1.34% (based on available data for mothers who had samples at UCI), which is in line with larger studies of colonization among the general population which is typically around 1%.^19,20^

The care provided by NICU personnel often requires them to lean over bassinets and work with small devices. This uniquely brings staff members faces in close proximity to a baby’s body and could result in transfer of MRSA from the nose, the major reservoir for *S. aureus*, to the baby.^5,21,22^ This transfer would be mitigated by mask wearing. In addition, masking has been shown to reduce face touching both among the public^23^ and healthcare personnel^24^ and may have further minimized the spread of MRSA from healthcare providers hands and skin as well as nose.

Reduction in MRSA acquisition during the period of universal masking raises the question of whether NICU staff should mask during patient care. This remains a controversial topic following the end of the COVID-19 pandemic with ongoing concerns that masks could stifle infant development, including emotional processing^25,26^ and language acquisition.^27^ There is also the concern that staff masking may impede communication between healthcare personnel and families.^28^ Finally, staff may find masking uncomfortable and obstructive. Nevertheless, the marked 56% reduction in MRSA acquisition is an important finding that could stave off serious morbidity and even mortality in this population, and perhaps masking during prolonged close contact “nose-to-nose” or “face-to-face” care should be adopted, as has been previously suggested.^11^

In contrast, MSSA may have been more frequently acquired from skin-to-skin contact with parents, where the infant is placed directly onto a parents’ bare chest for extended periods of time. This beneficial practice fosters bonding, stabilizes temperature, and promotes breastfeeding, especially in preterm infants.^29^ Skin-to-skin contact was actively encouraged during the pre-pandemic and pandemic periods of this study. While NICU healthcare providers did interact and pick up babies, they had high hand hygiene adherence before and after contact, and were expected to use physical barriers (clean blankets, towels) when holding babies to avoid non-hand skin-to-skin contact.

Both MRSA and MSSA acquisition pose a high risk of invasive infection in the NICU population due to an immunocompromised state from preterm birth, an immature skin barrier, high prevalence of invasive devices, and long hospital admissions. Thus, while *S. aureus* is a normal human commensal typically acquired from parents, the high-risk NICU setting may demand greater protection from this pathogen.

We found a strong association between maternal MSSA carriage and neonatal acquisition of the same. Maternal MRSA status did not reach this same degree of significance. It is not surprising that maternal flora plays a significant role in shaping the neonate’s microbiome, both inside and outside the NICU, as shown in previous cohort studies.^30,31^ Thus, there may be a benefit to parental decolonization strategies for neonates in critical care,^10^ including targeted nasal decolonization of colonized parents or use of chlorhexidine gluconate wipes to clean exposed parental skin prior to skin-to-skin contact. This practice could reduce *S. aureus* transmission from parent to infant without disrupting the benefit of skin-to-skin care since the major benefit of skin-to-skin parent-baby activities is physiologic rather than related to the neonatal microbiome.

This study was possible due to standard admission and weekly *S. aureus* screening performed for all babies within the evaluated NICU. Additionally, our hospital’s standard laboratory practice is to plate screening swabs on both MRSA chromogenic agar as well as blood agar to enhance MRSA detection. This allows for MSSA detection to be routinely recorded as well. This uniform active surveillance and laboratory identification protocol provided us with comprehensive information about *S. aureus* colonization to assess acquisition rates for both MRSA and MSSA.

Limitations of our study included an overall low incidence of MRSA, incomplete maternal *S. aureus* colonization data (only the birth mother was screened and maternal status was only known for inborn babies, and those whose mother had a clinical sample in the one year prior to neonatal NICU admission), no colonization data for other household members or visitors, and unknown *S. aureus* colonization status among NICU healthcare personnel during the period of study. We did not measure adherence to contact precautions for patients with MRSA in either period or frequency or duration of skin-to-skin time. We are also unable to isolate the effect of limiting visitors to the NICU during the pandemic period which may have affected exposure of neonates to both MRSA and MSSA.

## Conclusions

Our data demonstrated a significant reduction in risk for neonatal MRSA acquisition associated with universal masking during the pandemic masking period. Thus, provider masking for close contact activities between NICU staff and babies may mitigate transmission of this important pathogen. There was also a significantly increased risk of MSSA acquisition in neonates who had a mother with positive MSSA culture in the year prior to delivery. This suggests that parental decolonization may also protect these vulnerable patients from acquisition of *S. aureus* colonization and subsequent infection.
